# Oxidative Stress and the Pathogenesis of Alzheimer's Disease

**DOI:** 10.1155/2013/316523

**Published:** 2013-07-25

**Authors:** Yan Zhao, Baolu Zhao

**Affiliations:** ^1^Department of Bioengineering, Harbin Institute of Technology at Weihai, Weihai, Shandong 264209, China; ^2^State Key Laboratory of Brain and Cognitive Science, Institute of Biophysics, Chinese Academy of Sciences, Beijing 100101, China

## Abstract

Alzheimer's disease (AD) is the most common neurodegenerative disease that causes dementia in the elderly. Patients with AD suffer a gradual deterioration of memory and other cognitive functions, which eventually leads to a complete incapacity and death. A complicated array of molecular events has been implicated in the pathogenesis of AD. The major pathological characteristics of AD brains are the presence of senile plaques, neurofibrillary tangles, and neuronal loss. Growing evidence has demonstrated that oxidative stress is an important factor contributing to the initiation and progression of AD. However, the mechanisms that lead to the disruption of redox balance and the sources of free radicals remain elusive. The excessive reactive oxygen species may be generated from mechanisms such as mitochondria dysfunction and/or aberrant accumulation of transition metals, while the abnormal accumulation of Abeta and tau proteins appears to promote the redox imbalance. The resulted oxidative stress has been implicated in Abeta- or tau-induced neurotoxicity. In addition, evidence has suggested that oxidative stress may augment the production and aggregation of Abeta and facilitate the phosphorylation and polymerization of tau, thus forming a vicious cycle that promotes the initiation and progression of AD.

## 1. Introduction

Alzheimer's disease (AD) is the most common neurodegenerative disease that causes dementia in the elderly. It is characterized by the gradual deterioration of memory and other cognitive functions, which eventually leads to a complete incapacity and death of the patients within 3 to 9 years after diagnosis [1]. Increasing age is a major risk factor for sporadic forms of AD. As the elderly population of the world continues to increase, the prevalence of AD has increased remarkably worldwide, and AD has become one of the leading causes of disability and death among the elderly [2–5]. Despite the tremendous progress that has been made in AD research in the past few decades, the exact cause and pathogenesis of AD are not completely understood, and currently, there is no effective treatment for the disease. 

The major pathological characteristics of AD brains are the presence of senile plaques, neurofibrillary tangles (NFTs), and neuronal loss [1, 6]. Senile plaques are mainly composed of beta-amyloid peptide (Abeta) that is produced from proteolytic cleavage of the transmembrane amyloid precursor protein (APP). NFTs are formed by arrays of paired helical filaments (PHFs) structures, which contain mainly self-aggregated hyperphosphorylated tau, a multifunctional protein involved in microtubule assembly and stabilization [6]. Accumulating evidence has shown that the presence of extensive oxidative stress is a characteristic of AD brains in addition to the established pathology of senile plaques and NFT [7]. It has been demonstrated that the levels of protein carbonyls and 3-nitrotyrosine, which are resulted from protein oxidation, and markers of oxidative damage to DNA and RNA, such as 8-hydroxydeoxyguanosine (8-OHdG) and 8-hydroxyguanosine, are elevated in AD brains [8–12]. Products of lipid peroxidation, such as malondialdehyde (MDA), 4-hydroxynonenal, and F_2_-isoprostanes, are also increased in multiple brain regions and cerebrospinal fluid (CSF) of patients with AD or mild cognitive impairment (MCI) [13–17]. In addition to the accumulation of free radical damage, alterations in the activities or expression of antioxidant enzymes such as superoxide dismutase (SOD) and catalase have been observed in both central nervous system and peripheral tissues of AD patients [17–20]. Moreover, in AD and MCI brains, the increased oxidative damage to lipids and proteins and the decline of glutathione and antioxidant enzyme activities are more localized to the synapses and correlate with the severity of the disease, suggesting an involvement of oxidative stress in AD-related synaptic loss [21]. Importantly, many of the previously mentioned studies show elevations of oxidative stress in MCI, which is proposed as an intermediate state between normal aging and dementia, indicating that the oxidative stress damage in AD may occur preceding the onset of the disease. These results suggest that oxidative stress may be one of the earliest alterations that occur during the initiation and development of AD. 

While oxidative stress has emerged as one of the important factors in AD pathogenesis, the mechanisms by which the redox balance is altered and the sources of free radicals remain elusive. The present paper reviews the involvement of abnormal accumulation of Abeta and tau proteins in the induction of redox imbalance and the generation of free radicals through mechanisms such as mitochondria dysfunction and/or transition metal homeostasis imbalance and discusses the mechanisms by which oxidative stress promotes Abeta- and tau-mediated neurotoxicity.

## 2. Oxidative Stress and Abeta-Induced Toxicity

Abeta is produced via sequential proteolytic cleavages of APP by two membrane-bound proteases, beta-secretase, also known as beta-site APP cleaving enzyme 1 (BACE1), and gamma-secretase, a multiprotein complex consisting of presenilin (PS), nicastrin (NCT), anterior pharynx-defective 1 (APH-1), and presenilin enhancer protein 2 (PEN-2) [1, 6, 22]. BACE1 cleaves APP at the N-terminal end, producing a 99 amino acid APP C-terminal fragment, which is further cleaved within the transmembrane domain by gamma-secretase, resulting in the release of Abeta peptides [1, 6, 23]. Several peptides of varying lengths can be generated from the cleavages by beta- and gamma-secretases; among them, the 42-amino acid form of Abeta (Abeta42) is more toxic than the more abundantly produced 40-amino acid form of Abeta (Abeta40), possibly because of its faster self-aggregation into oligomers [1, 23, 24]. In fact, multiple lines of evidence suggest that soluble Abeta oligomers are the most neurotoxic, whose levels correlate with the severity of the cognitive decline in AD [23, 24]. Cleavage of APP by a third enzyme, alpha-secretase, precludes the formation of toxic Abeta peptides [25]. Increased production and/or decreased clearance of Abeta peptides leads to the accumulation of Abeta, which stimulates diverse cell signaling pathways, eventually resulting in synaptic degeneration, neuronal loss and decline in cognitive function [6, 23, 24, 26–28]. 

A great deal of research has implicated oxidative stress in Abeta-induced neurotoxicity [29]. *In vitro *experiments using cell models showed that Abeta treatment could increase the levels of hydrogen peroxide and lipid peroxides [30]. Consistently, in various AD transgenic mouse models carrying mutants of APP and PS-1, increased hydrogen peroxide and nitric oxide production as well as elevated oxidative modifications of proteins and lipids were correlated with the age-associated Abeta accumulation, confirming that Abeta promotes oxidative stress [31–35]. In hippocampal neuronal cell cultures, the induction of reactive oxygen species (ROS) by soluble Abeta oligomers required the activation of N-methyl-D-aspartate (NMDA) receptor and was associated with a rapid increase in neuronal calcium levels, suggesting a possible role of soluble Abeta oligomers as proximal neurotoxins and the involvement of oxidative stress in the synaptic impairment and neuronal loss induced by soluble Abeta oligomers [36]. Consistently, it has been demonstrated in AD cell and animal models that natural antioxidants, such as EGb 761, curcumin, and green tea catechins, can exert neuroprotective functions by attenuating Abeta-induced ROS generation and neuronal apoptosis [37–40].

In addition to mediating Abeta-induced cytotoxity, numerous studies have suggested that oxidative stress promotes the production of Abeta. It was demonstrated that defects in antioxidant defense system caused elevated oxidative stress and significantly increased Abeta deposition in transgenic mice overexpressing APP mutant [41, 42], while dietary antioxidants such as curcumin lowered the elevation of oxidized proteins and decreased brain Abeta levels and Abeta plaque burden [43]. Moreover, the increased Abeta deposition and its associated earlier onset and more severe cognitive dysfunction induced by the defect in antioxidant defense system could be ameliorated by antioxidant supplementation [42]. In line with these findings, overexpression of manganese superoxide dismutase (MnSOD) in Tg19959 transgenic mice overexpressing APP mutant decreased protein oxidation and increased antioxidant defense capability in brains while reducing Abeta plaque burden and restoring the memory deficit [44]. Furthermore, deletion of cytoplasmic copper/zinc superoxide dismutase (Cu-Zn-SOD, SOD1) in Tg2576 APP-overexpressing AD mouse model was found to increase Abeta oligomerization while accelerating the loss of spatial learning and memory as compared with control AD mice, suggesting a possible role of oxidative damage in Abeta oligomerization [45]. These results suggest that the enhancement of Abeta production/plaque formation as well as Abeta oligomerization by oxidative stress is important for the initiation and development of AD. 

Studies on how oxidative stress enhances Abeta production have revealed that oxidative stress decreases the activity of alpha-secretase while promoting the expression and activation of beta- and gamma-secretase, enzymes critical for the generation of Abeta from APP [46–50]. The induction of BACE1 and PS1 expression and the activation of gamma-secretase by oxidative stress were found to be dependent on the activation of c-Jun N-terminal kinase (JNK) pathway, a major cell signaling cascade that is stimulated by oxidative stress [51, 52]. In fact, the promoter and 5′ untranslated region of BACE gene contain binding sites for multiple transcription factors including the redox-sensitive activator protein (AP1) and nuclear factor (NF)-kappa B, activation of which by oxidative stress may in turn enhance BACE expression [53]. In AD brains, both the activation of JNK signaling cascade [54–56] and the elevation of BACE1 and PS1 expression/activity have been detected [57–59]; thus, it is possible that the increased oxidative stress in AD brains may initiate the activation of a cascade of redox-sensitive cell signal pathways including JNK, which promotes the expression of BACE1 and PS1, eventually enhancing the production of Abeta and the deterioration of cognitive function. As JNK has also been implicated in Abeta-induced neuronal apoptosis [60], oxidative stress may enhance Abeta production as well as mediate Abeta-induced neurotoxicity through the activation of redox-sensitive signaling pathways such as JNK.

Alternatively, the augmentation of Abeta production by oxidative stress may be a compensatory reaction to oxidative stress. It was found that neuronal oxidative damage was more pronounced in AD subjects with lesser amounts of Abeta deposition or in AD subjects with a shorter disease duration [61, 62], and there was an inverse relationship between the levels of neuronal oxidative damage to nucleic acids and the amounts of intraneuronal Abeta42 in the hippocampus and the subiculum of AD brains [63]. These unexpected observations have led to the hypothesis that Abeta may potentially play a protective role against neuronal oxidative stress [64]. Indeed, evidence has suggested that picomolar or low nanomolar levels of Abeta can be neurotrophic or neuroprotective [65, 66]. Physiological concentrations of Abeta were shown to efficiently inhibit autooxidation of lipoproteins in CSF and plasma [67] and markedly increase hippocampal long-term potentiation [68], whereas the high nanomolar concentrations of Abeta caused the well-established toxic effects. In addition, it appeared that the dualistic effects of Abeta depended on the aggregation state of Abeta and were Abeta size-form specific [66, 69]. Taken together, low levels of Abeta may have a role in the normal function of neuronal cells and could beneficially influence the cellular redox status, while the abnormal accumulation and aggregation of specific forms of Abeta, which can be enhanced by oxidative stress, may impair neuronal function and further exacerbate neuronal oxidative damage, contributing to the pathological development of AD. A better understanding of the pathological as well as the physiological role of Abeta may lead to more effective strategies for AD interventions.

## 3. Mitochondria Dysfunction and Oxidative Stress in AD 

Mitochondria are unique organelles that are pivotal for a variety of cellular functions including ATP synthesis, calcium homeostasis, and cell survival and death. Meanwhile, mitochondrial respiratory chain is a major site of ROS production in the cell, and mitochondria are particularly vulnerable to oxidative stress [70, 71]. Extensive studies have demonstrated that mitochondria dysfunction is an important factor involved in the pathogenesis of AD. A number of mitochondrial and metabolic abnormalities have been identified in the hippocampal neurons of AD compared to age-matched controls [72–74]. Morphometric analysis of biopsies from AD brains showed a significant reduction of mitochondria, while the mitochondrial DNA and protein were increased in the cytoplasm and in the vacuoles associated with lipofuscin, a lysosome suggested as the site of mitochondrial degradation by autophagy [72, 73]. These mitochondrial abnormalities were found accompanied by oxidative damage marked by 8-hydroxyguanosine and nitrotyrosine, indicating that the mitochondria were damaged during the progression of AD [72]. In line with this, a significant decrease of mitochondrial cytochrome oxidase (complex IV) activity in the cortical regions of AD brains was reported [74]. Deficiency in this key electron transport enzyme could lead to the increase in ROS production and reduction in energy stores, eventually contributing to the neurodegenerative process [74].

Evidence suggests that Abeta may directly disrupt mitochondria function and contribute to the deficiency of energy metabolism and neuronal death seen in AD. It was found that Abeta was localized to mitochondria in brains of AD patients and transgenic mice as well as in neuroblastoma cells stably expressing human mutant APP [34, 75]. The presence of Abeta in mitochondria was associated with impaired mitochondrial metabolism and increased mitochondrial ROS production [34, 75]. In fact, in isolated mitochondria, Abeta treatment could cause oxidative injury to mitochondrial membrane, disrupt lipid polarity and protein mobility and inhibit key enzymes of the mitochondria respiratory chain, leading to increased mitochondrial membrane permeability and cytochrome c release [76, 77]. MnSOD, a primary antioxidant enzyme protecting mitochondria against superoxide, was found to be a target of nitration and inactivation in a double homozygous knock-in mouse model expressing APP and PS-1 mutants [78]. The decreased activity of antioxidant defense enzymes such as MnSOD may further increase ROS levels and compromise mitochondria function, contributing to the loss of mitochondrial membrane potential and eventually caspase activation and apoptosis [78]. Abeta has also been shown to alter other cellular protective mechanisms against oxidative damage to mitochondria. Uncoupling proteins (UCPs) are a family of mitochondrial anion carrier proteins that are located on the inner mitochondrial membrane with diverse physiological functions [79]. It has been demonstrated that UCP2 and UCP3 can be activated by ROS or products of lipid peroxidation to diminish proton motive force and reduce mitochondrial membrane potential and ATP production, causing mitochondria uncoupling and decrease of ROS generation from mitochondria [80]. Therefore, the expression and activation of UCPs are considered to be a protective mechanism in response to oxidative stress. This protective mechanism appears dysfunctional in AD brains where the expression of UCP2, 4, and 5 is significantly decreased [81]. In SH-SY5Y neuroblastoma cells overexpressing APP or APP mutant, it was found that the upregulation of UCP2 and UCP4 protein levels in response to the exposure of superoxide was abrogated; although the mechanisms are unclear, it suggests that Abeta accumulation may lead to irreversible cellular alterations that render the cell more susceptible to oxidative stress [82]. Moreover, the UCP2- and UCP4-dependent upregulation of mitochondrial free calcium in response to superoxide treatment was found to be diminished in cells overexpressing APP or APP mutant, indicating that the Abeta accumulation may be associated with a dysfunction of mitochondria as a reserve pool of intracellular calcium that leads to an increased cell sensitivity to the loss of calcium homeostasis [82]. 

It is noted that the mitochondria-associated Abeta along with the increase in hydrogen peroxide and decrease in cytochrome oxidase activity was detected prior to the appearance of Abeta plaques, suggesting the defect in mitochondria occurs earlier in the pathogenesis of AD. Therefore, early mitochondrially targeted therapeutic interventions may be effective in delaying the onset and progression of AD [34, 75].

## 4. Metal Homeostasis and Oxidative Stress in AD

Transition metals such as copper (Cu), zinc (Zn), and iron (Fe) play important catalytic roles in many enzymes and are essential for a broad range of biological processes in human body including brain functions. Both Cu and Zn have been shown to participate in regulating synaptic function. Following NMDA receptor activation, Cu is released from the neuron and regulates neuronal activation by functionally blocking NMDA receptors and limiting calcium entry into the cell [83]. Zn also has a neuromodulatory role; it is released from presynaptic nerve terminals into the synaptic cleft upon neuronal activation and has been shown to inhibit excitatory NMDA receptors [84]. Fe is crucial for neuronal processes such as myelination, synaptogenesis, and synaptic plasticity (SP). It is well documented that deficiency of Fe can induce a series of neurochemical alterations that may eventually lead to cognitive deficits [85]. While these transition metals play essential roles in neural functions, their levels and transport are strictly regulated, as aberrant metal homeostasis can result in neurotoxic free-radical production. For example, excess Fe or Cu can directly interact with oxygen to produce superoxide ion, hydrogen peroxide, and hydroxyl radical, which may lead to oxidative stress and a cascade of biochemical alterations that eventually cause neuronal cell death [86]. In fact, growing evidence has shown that there is a close relationship between the disruption of metal homeostasis and AD [86]. Abnormal levels of Cu, Zn, and Fe have been observed in AD hippocampus and amygdala, areas showing severe histopathologic alterations [87]. Moreover, these transition metals have been detected within the amyloid deposits in AD patients as well as transgenic mouse models [88–90]. These data suggest that the aberrant accumulation of transition metals may be inextricably linked with Abeta pathology in AD; however, the precise cause and the nature of the involvement of brain metal dyshomeostasis in AD are still largely unknown. 

The presence of transition metals within the amyloid deposits in AD patients indicates that transition metals may directly interact with Abeta [88–90]. Indeed, both Cu^2+^ and Zn^2+^ can bind to Abeta monomers via three histidine residues (His^6^, His^13^, and His^14^) and a tyrosine residue (Tyr^10^), causing conformational changes of the peptide that promote its aggregation [91, 92]. Consistently, *in vitro* data showed that Cu and Zn rapidly induced the aggregation of soluble Abeta peptides [93, 94]. Therefore, the disturbance of metal homeostasis and the abnormal interactions of Abeta with metal ions may be directly involved in the process of Abeta deposition in AD brains [93–96]. In addition, the aberrant interaction between transition metals and Abeta may be a source of ROS generation. Abeta binds Cu^2+^ with high affinity, forming a cuproenzyme-like complex [91]. Electrons can be transferred from Abeta to Cu, reducing Cu^2+^ to Cu^+^ and forming positively charged Abeta radical (Abeta^+•^) [97]. Cu^+^ then donates two electrons to oxygen, generating H_2_O_2_ [97, 98], setting up conditions to further produce hydroxyl radicals (Fenton-type reaction) [99, 100]. After electron donation to O_2_, the radicalized ·Cu^2+^ complex may be restored to Abeta·Cu^2+^ by electron transfer from biological reducing agents such as cholesterol, catecholamines, and vitamin C [97]. The efficiency for generation of H_2_O_2_ is greater for Abeta42 than Abeta40, correlating with their cytotoxic activity [98, 99]. Similar to copper-Abeta interaction, binding of Fe to Abeta results in reduction of Fe^3+^ to Fe^2+^ and the generation of H_2_O_2_ [101]. These data suggest that ROS generated from the interaction of transition metals with Abeta are key contributors to the oxidative stress in Abeta-mediated neurotoxicity and AD pathogenesis. 

Additionally, there is a close association between Fe/Cu homeostasis and the production and processing of APP. In SH-SY5Y cells overexpressing the Swedish mutant form of human APP (APPsw), Fe treatment induced the release of Abeta42 [102, 103]. Moreover, an iron-responsive element (IRE-Type II) was identified within the 5′-untranslated region (5′-UTR) of APP transcript, which was selectively downregulated in response to intracellular Fe chelation; thus, in addition to promotion of Abeta generation from APP, increases in Fe levels may lead to upregulation of APP protein translation through binding of Fe regulatory proteins to the APP IRE [104]. On the contrary, Cu treatment was shown to promote the nonamyloidogenic pathway of APP and decrease the release of Abeta [105]. This may be related to the finding that Cu is abnormally distributed in AD brain, with accumulation of Cu in amyloid plaques but a deficiency of Cu in neighboring cells [87, 88]. 

Studies have shown that metal transporters such as Zn transporters and divalent metal transporter 1 (DMT1) are increased in the cortex and hippocampus of APP/PS1 transgenic mice; and similar to transition metals, these metal transporters are colocalized with Abeta in senile plaques in the cortex of AD brains [103, 106, 107]. This leads to the speculation that metal transporters may play important roles in the aberrant metal homeostasis in AD. DMT1, also known as natural resistance-associated macrophage protein 2 (Nramp2) or divalent cation transporter 1 (DCT1), is a newly discovered proton-coupled metal-ion transport protein responsible for the uptake of a broad range of divalent metal ions, including Fe, Cu, and Zn [108]. In APPsw cells, where a significant increase in DMT1 levels was found when compared to the control cells [103], it was observed that the intracellular Fe was significantly elevated along with the increased oxidative stress and cell toxicity [102, 103]. Silencing of endogenous DMT1 by RNA interference (RNAi) decreased the protein levels of DMT1 and also reduced bivalent ion influx into the cells, suggesting that the elevation of DMT1 might be involved in the disruption of Fe homeostasis seen in APPsw cells [102, 103]. 

APP, which has a Cu binding site at N-terminal cysteine-rich region [109], generates free radicals when interacting with Cu. In addition, APP has been shown to modulate Cu homeostasis. Overexpression of APP in Tg2576 transgenic mice caused a significant reduction in Cu levels [110]. In APP knockout mice, Cu levels were significantly elevated in cerebral cortex, a region of the brain particularly involved in AD [111, 112]. This leads to the speculation that the secreted APP and/or Abeta may promote the efflux of Cu or prevent its uptake, thus reducing its levels [110]. In SH-SY5Y cells, it was found that endogenous APP had a partial colocalization with the Golgi marker GM130, while the elevation of cellular Cu levels promoted the exit of APP from the Golgi to a wider distribution throughout the cytoplasm and to the plasma membrane [113]. It was suggested that the increase in cell surface APP was resulted from a concomitant increase in exocytosis and reduction in endocytosis [113]. The copper-responsive trafficking of APP is therefore consistent with a role for APP in Cu efflux pathways [111, 113]. These observations suggest that there is an interdependent relationship between APP metabolism and Cu homeostasis, perturbations of either may cause alteration of the other, eventually promoting the accumulation of Abeta and the generation of free radicals [112]. 

As aberrant metal homeostasis plays an important role in several important aspects of AD pathogenesis including the production and aggregation of Abeta and the oxidative stress mediated by Abeta, AD therapy-targeted metal-Abeta interaction is rapidly emerging as a promising therapeutic option. Various metal chelating compounds have been tested for their efficacy [114]. One of these compounds, clioquinol (CQ), an orally bioavailable Cu/Zn chelator, was shown to block Abeta-induced production of H_2_O_2_ and Zn^2+^/Cu^2+^-induced precipitation of synthetic Abeta *in vitro* and significantly reduce the level of Abeta deposition in brains of Tg2576 transgenic mice [96, 115]. In a randomized, double-blind, placebo-controlled clinical intervention using clioquinol in patients with moderately severe AD, CQ treatment lowered plasma Abeta42 levels and slowed cognitive impairment in the more severely affected patients (baseline cognitive subscale score of the AD Assessment Scale, ≥25) during a 36-week period [116]. 

Since the generation of ROS is an important factor in AD pathogenesis promoted by the aberrant accumulation of transition metals, natural antioxidants may have protective effects against AD pathogenesis induced by disruption of metal homeostasis [117]. As expected, treatment with antioxidant nicotine attenuated the copper-facilitated neurotoxicity induced by Abeta in APPsw cells while decreasing the intracellular Cu concentration [90]. Consistently, nicotine treatment significantly lowered the Cu and Zn concentrations in senile plaques and a subfield of the hippocampus CA1 region in the brains of APPV717I (London mutant form of APP) transgenic mice, and these effects were found to be independent of the activation of nicotinic acetylcholine receptors [90]. These results suggest that antioxidants such as nicotine may also have a role in regulating metal homeostasis. 

## 5. Oxidative Stress and Tau Pathology

Hyperphosphorylated tau protein is the major component of NFT, another hallmark of AD pathology that correlates with neurodegeneration and cognitive decline [6]. Abnormal hyperphosphorylation of tau impairs its binding with tubulin and its capacity to promote microtubule assembly, resulting in its self-aggregation into filaments [118]. A number of protein kinases and protein phosphatases have been implicated in the abnormal phosphorylation of tau including glycogen synthase kinase-3 beta (GSK-3 beta), cyclin-dependent kinase 5, mitogen-activated protein kinase (MAPK), calcium-calmodulin kinase, and protein kinase C [119]. It has been suggested that the accumulation of Abeta may appear before the tau pathology and that Abeta aggregates may be one of a cascade of molecular events leading to tau hyperphosphorayltion [120–122]. On the other hand, it was reported that overexpression of tau inhibited kinesin-dependent transport of peroxisomes, neurofilaments, and Golgi-derived vesicles into neurites, causing transport defects in primary neuronal cells including the trafficking of APP [123]. In particular, the transport of APP into axons and dendrites was blocked, causing its accumulation in the cell body [123]. 

Although less well studied, evidence has shown that oxidative stress is interlinked with tau pathology. It was also shown that the cells overexpressing tau protein had increased susceptibility against oxidative stress, perhaps due to the depletion of peroxisomes [123]. In a drosophila model of human tauopathies expressing a disease-related mutant form of human tau (tau R406W), reduction of gene dosage of thioredoxin reductase (TrxR) or mitochondrial SOD2 enhanced tau-induced neurodegenerative histological abnormalities and neuronal apoptosis [124]. In contrast, overexpression of these antioxidant enzymes or treatment with vitamin E attenuated tau-induced neuronal cell death [124]. Moreover, in cortical neurons derived from a transgenic rat model expressing a human truncated variant form of tau protein, it was observed that the levels of ROS were increased when compared to control nontransgenic neurons, while antioxidants such as vitamin C significantly eliminated the elevation of ROS [125, 126]. These observations suggest that tau-induced neurotoxicity is at least partially mediated by oxidative damage [124]. The linkage between oxidative stress and tau pathology was further demonstrated in P301S and P301L transgenic mouse models carrying the human tau gene with P301S or P301L mutations, which exhibit an accumulation of hyperphosphorylated tau and develop neurofibrillary tangles and neurodegeneration [127]. Mitochondrial dysfunction together with reduced NADH-ubiquinone oxidoreductase activity was found in P301L tau transgenic mice, which was associated with increased ROS production, impaired mitochondrial respiration and ATP synthesis in aged animals [128]. Similarly, the brains of P301S transgenic mice exhibited signs of elevated oxidative stress including increased protein carbonyl levels in cortex mitochondria, alterations in the activity and content of mitochondrial enzymes involved in ROS formation and energy metabolism, suggesting that oxidative stress and mitochondrial dysfunction might play an important role in tau pathology [129]. Consistently, administration of P301S mice with coenzyme Q10, an antioxidant and a key component of the electron transport chain, significantly increased complex I activity and reduced lipid peroxidation while improving survival and behavioral deficits of the mice [130]. Furthermore, the convergence of Abeta and tau pathologies on mitochondria dysfunction was demonstrated in a triple transgenic mouse model [pR5/APP/PS2] (^triple^AD), which exhibits both Abeta and tau pathologic features of the disease in the brain of the animal [131]. Proteomics analyses of the ^triple^AD brain samples demonstrated a massive deregulation of 24 proteins, of which one third were mitochondrial proteins mainly related to complexes I and IV of the oxidative phosphorylation system [132]. Notably, deregulation of mitochondrial complex IV was shown to be Abeta dependent, while deregulation of complex I was tau dependent [132]. The effects of Abeta and tau on mitochondrial function were found to be synergistic and age associated, resulting in the decrease of the mitochondrial respiratory capacity and the reduction of ATP synthesis, which finally led to the synaptic loss, and neuronal death [132]. 

Growing evidence has also shown that oxidative stress may have a role in the hyperphosphoryaltion and polymerization of tau. The oxidation of fatty acids, which is found to be elevated in AD brains, was reported to facilitate the polymerization of tau, and thus might serve as a possible link between oxidative stress and the formation of the fibrillar pathology in AD [133]. In Tg2576 AD transgenic mice, deficiency in mitochondrial SOD2 [134] or reduction of cytoplasmic SOD1 induced tau phosphorylation, suggesting that ROS may play a critical role in the hyperphosphoryaltion of tau [45]. p38 MAPK, which can be activated by oxidative stress, is capable of phosphorylating tau protein *in vitro* [135]. In hippocampal and cortical brain regions of AD patients, activated p38 is found exclusively localized to NFT and coimmunoprecipitated with PHF-tau, suggesting that it might be involved in the phosphorylation of tau *in vivo* [136]. Thus, p38 may be a candidate that links the phosphorylation of tau with increased oxidative stress in AD.

## 6. Conclusions

In summary, evidence has demonstrated that oxidative stress is inextricably linked with several major pathological processes in AD including Abeta-induced neurotoxicity, tau pathology, mitochondria dysfunction, and metal dyshomeostasis. Excessive ROS may be generated from mitochondria dysfunction and/or aberrant accumulation of transition metals, perhaps caused by a combination of abnormal Abeta accumulation and tau pathology, eventually resulting in oxidative stress. Oxidative stress, which mediates the neurotoxicity induced by abnormal accumulation of Abeta and tau proteins, may augment Abeta production and aggregation as well as facilitate tau phosphorylation and polymerization, further enhancing a variety of neurotoxic events including ROS production, thus forming a vicious cycle that promotes the initiation and progression of AD. Regardless a primary or secondary event, oxidative stress is an important factor contributing to the development of AD. Removal of ROS or prevention of their formation may delay the onset or slow down the progression of AD through multiple mechanisms including, but not limited to, reduction of oxidative stress-mediated neuronal toxicity, inhibition of Abeta production and aggregation, decrease of tau phosphorylation and polymerization, and restoration of mitochondria function and metal homeostasis. Therefore, AD prevention or treatment with natural antioxidant may be an approach that is capable of targeting a number of different molecular events implicated in the pathogenesis of AD. 
